# 25-years Trends and Risk factors related to Surgical Outcomes of Giant Retinal Tear-Rhegmatogenous Retinal Detachments

**DOI:** 10.1038/s41598-020-61592-0

**Published:** 2020-03-25

**Authors:** Daniel S. W. Ting, Valencia H. X. Foo, Tien-En Tan, Nicole M. Sie, Chee Wai Wong, Andrew S. H. Tsai, Gavin S. W. Tan, Laurence S. Lim, Ian Y. S. Yeo, Doric W. K. Wong, Sze Guan Ong, Edmund Y. M. Wong, Chong Lye Ang, Shu Yen Lee

**Affiliations:** 1 Singapore National Eye Centre, Singapore Eye Research Institute, Singapore, Singapore; 2 Duke-National University of Singapore Medical School, Singapore, Singapore

**Keywords:** Eye abnormalities, Vitreous detachment

## Abstract

To describe the 25-year surgical trends, long-term outcomes and risk factors affecting the outcomes of giant retinal tear-related rhegmatogenous retinal detachments (GRT-RRD). Patients’ demographics, pre-operative characteristics, risk factors, operative procedures and post-operative outcomes were collected and divided into three groups – Group A: 1991 to 2015 (overall); Group B: 1991 to 2005, and Group C: 2006 to 2015. Functional and anatomical successes were monitored over a 5-year period. Multivariate logistic regression analysis was performed to identify the risk factors related to functional and anatomical success.127 eyes of 127 patients were included in the study. At 5^th^ year, 69.4% patients had visual acuity (VA) < logMAR 1.0 with 87.5% primary anatomical success rate. While the functional outcome remained the same between group B and C, there was an increase in the anatomical success from 89.7% to 100%, albeit not statistically significant. Patients with worse presenting VA, 150 degrees or more of giant retina tear, macula-detached status and presence of PVR were associated with VA of> logMAR 1.0 (all p < 0.05). The types of surgery (TPPV vs combined SB/TPPV), number of breaks, lens extraction and additional cryotherapy were not associated with the functional or anatomical success. In conclusion, the GRT-RRD functional and structural outcomes were comparable between 1991–2005 and 2006–2015, albeit a statistically insignificant improvement of anatomical outcome over the past 25 years. Worse presenting VA, 150 degrees or more of giant retinal tear, detached macula and presence of PVR were associated with poorer visual outcome.

## Introduction

A giant retinal tear (GRT) is defined as a full-thickness retinal break of at least 90° in circumferential extent^1–3^. In United Kingdom, the incidence of GRTs are rare, with an estimation of 0.094 cases per 100,000 per year^2^. Surgical management of GRT-related rhegmatogenous retinal detachments (GRT-RRD) is challenging, and often complicated by re-detachment and the development of proliferative vitreoretinopathy (PVR). Many refinements in vitreoretinal surgical instruments occurred over the past 15 years, including the introduction of smaller-gauge port vitrectomies, faster-speed cutters, wide-angle illumination system, perfluorocarbon liquid and chandelier lights^4^. These instruments and technologies have enhanced surgeons’ ability to thoroughly evaluate the peripheral retina with higher magnification and better illumination in detecting retinal breaks and detecting vitreoretinal traction^5^. These technical developments have been associated with improved rates of anatomical success rates reported in the literature^3,6,7^.

The surgical options for GRT repair include primarily trans pars plana vitrectomy (TPPV) with or without the combined use of an encirclage scleral buckle (SB) or primary scleral buckle (with segmental/encirclage SB). It is thought that the addition of an SB may help reduce vitreoretinal tractional forces, although this is controversial; some surgeons instead believe that an SB can cause the posterior retinal slippage of GRT due to the change in ocular contour and sclera shortening relative to the retina. Regardless of the choice of surgical methods, anatomical success rates range from 86% to 94%^2,4,8–11^. Lee *et al*. reported an anatomical success rate of 85% and an improvement of visual acuity VA) of 20/40 or better in 40% after at least 6 months of follow-up^10^, with all patients undergoing TPPV (100%) and additional SB performed for 70.3% for all GRT-RRD patients. Subsequently, Ang *et al*. reported a good outcome on primary TPPV for 62 GRT-RRD patients in UK, with laser retinopexy and silicone oil tamponade^2^, with 94.7% anatomical success and 42.1% functional success (VA > 20/40 or better).

To date, few studies have reported on the long-term functional and anatomical outcomes, with the risk factors associated with the post-operative success rate. Thus, our study aims to report the 25-year GRT-RRD management trend, surgical outcomes and identification of the pre-operative factors associated with the post-operative outcomes that could affect the final functional or anatomical success, using a large 25-year cohort of GRT-RRD patients in a large tertiary eye centre.

## Methods

This was a retrospective study including all patients who underwent surgical management of GRT-RRDs at the Singapore National Eye Centre over a 25-year period from January 1991 to December 2015. This study was approved by the SingHealth institutional review board with exemption of patients’ consent, adhering to the tenets of Declaration of Helsinki.

The case records of all patients with GRT-RRD were extracted from the electronic medical system. Of the 175 eyes, 168 eyes met the definition of GRT-RRD, defined as 90 degrees or more retinal tear with neuro-sensory detachment of the retina. Of those, 136 eyes had at least a complete 1-year data, with 9 patients having bilateral GRT-RRDs. For data analysis, we utilized 1 eye from each patient and thus, 127 eyes were included in the final analysis for 1-year analysis. Of those, 72 eyes had 5-year functional and structural outcome that were utilized for long-term outcome assessment.

All patient demographics, pre-operative baseline characteristics, surgical technique, and post-operative outcomes were collected. Pre-operative data included patients’ demographics, best-corrected visual acuity (BCVA) at presentation, refractive errors, lens status, circumferential extent of GRT-RRD, number of tears (single/multiple), macula status (attached/partially detached/totally detached), and the presence of any primary PVR. Macula status was termed “attached” if the entire macula was attached, “totally detached” if the entire macula was detached, and “partially detached” if only part of the macula was detached. Intra-operative data included type of surgery performed, use of surgical adjuncts such as external drainage of subretinal fluid, perfluorocarbon liquids (PFCL), laser photocoagulation, lens removal, cryotherapy and the types of endotamponade used.

Post-operative outcomes included post-operative BCVA and retinal re-attachment rates for the first 5 years, post-operative complications and number of repeat retinal reattachments procedures (excluding procedures for removal of silicon oil alone). At each post-operative time-point, functional success was defined as a BCVA of <logMAR 1.0, primary anatomical success was defined as a 360° attached retina without any endo-tamponade with only a single retinal reattachment procedure (excluding removal of silicone oil) and final anatomical success was defined as having a 360° attached retina without any endo-tamponade, regardless of the number of retinal reattachment procedures required. Failure referred to a persistently detached retina. In order to evaluate the differences in the preferred practice patterns and outcomes, the 25-year cohort was divided into: Group A: between 1991 and 2015 inclusive; Group B: between 1991 and 2005 inclusive; and Group C: between 2006 and 2015 inclusive. These 3 groups were compared in terms of pre-operative, intra-operative, and post-operative characteristics.

### Statistical analysis

First, we showed the pre-, intra-operative and post-operative characteristics for all 3 groups, and analysed the differences between Groups B and C via chi-squared tests. Second, we analysed the relationships between various potential risk factors and the 12-month functional and anatomical outcomes, using multi-variate logistic regression analyses. Third, we compared the functional and anatomical outcomes between groups B and C over a 5-year period, using Kaplan-Meier survival curves. An appropriate Bonferroni correction (α/5) was applied for the 5-year follow-up outcomes to correct for the number of time-points evaluated resulting in a *P* value threshold of 0.01 to be considered statistically significant. All statistical analysis was performed using Stata Statistical computer package (STATA Statistical Software, Version 12, Statacorp, College Station, Texas, USA) and R (R Core Team, R Foundation for Statistical Computing, Vienna, Austria). The *p*-values of <0.05 in 2-sided tests was considered as statistically significant.

## Results

A total of 127 eyes of 127 patients were included in the study (Group A), with 78 patients in Group B and 49 patients in Group C. The mean age (standard deviation – SD) was 42.5 (+13.7) years, with male (n = 111, 87.4%) and Chinese predominance (n = 100, 78.7%) **(**Table 1**)**.

**Table 1:** Patient demographics and pre-operative characteristics of patients with giant retina tear-related rhegmatogenous retinal detachment, divided into two groups – 1991 to 2005 and 2006 to 2015.

Characteristics	Group A 1991 to 2015 (n = 127)	Group B - 1991 to 2005 (n = 78)	Group C - 2006 to 2015 (n = 49)
Patient demographics			
Age, mean (SD)	42.5 (13.7)	41.65 (12.5)	43.9 (15.5)
Gender, number (%)			
Male	111 (87.4%)	70 (89.7%)	41 (83.7%)
Female	16 (12.6%)	8 (10.3%)	8 (16.3%)
Race, number (%)			
Chinese	100 (78.7%)	66 (84.6%)	34 (69.4%)
Malay	8 (6.3%)	5 (6.4%)	3 (6.1%)
Indian	7 (5.5%)	3 (3.9%)	4 (8.2%)
Others	12 (9.5%)	4 (5.1%)	8 (16.3%)
Pre-operative characteristics			
LogMAR BCVA, mean (SD)	1.22 (1.0)	1.12 (0.8)	1.37 (1.3)
>1.0	62 (48.8%)	39 (50%)	23 (47.0%)
0.3 < x < = 1.0	37 (29.2%)	24 (30.8%)	13 (26.5%)
<= 0.3	28 (22.0%)	15 (19.2%)	13 (26.5%)
Pre-op refractive status, number (%)			
Highly myopic (SE < = −6.0D)	34 (26.8%)	19 (24.4%)	15 (30.6%)
Myopic (SE > −0.5, but < −6.0D)	82 (64.6%)	54 (69.2%)	28 (57.1%)
Non-myopic	11 (8.7%)	5 (3.9%)	6 (12.3%)
Lens status, number (%)			
Phakic	86 (67.7%)	53 (68.0%)	33 (67.4%)
Pseudophakic	30 (23.6%)	16 (20.5%)	14 (28.6%)
Aphakic	11 (8.7%)	9 (11.5%)	2 (4.1%)
Extent of GRT, number (%)			
90 to <120 degrees	66 (52.0%)	38 (48.7%)	28 (57.1%)
120 to <150 degrees	24 (18.9%)	15 (19.2%)	9 (18.4%)
150 to <180 degrees	28 (22.0%)	16 (20.5%)	12 (24.5%)
180 degrees or more	9 (7.1%)	9 (11.5%)	0 (0%)
Tears, number (%)			
Single	86 (67.7%)	60 (76.9%)	26 (53.1%)
Multiple	41 (32.3%)	18 (23.1%)	23 (46.9%)
Macula status, number (%)			
Attached	53 (41.7%)	30 (38.5%)	23 (46.9%)
Partially Detached	11 (8.7%)	5 (6.4%)	6 (12.2%)
Totally Detached	63 (49.6%)	43 (55.1%)	20 (40.8%)
Presence of primary PVR, number (%)			
Yes	17 (13.4%)	11 (14.1%)	6 (12.2%)
No	110 (86.6%)	67 (85.9%)	43 (87.8%)

^*^A two-sample t-test or Fisher’s exact test was conducted for continuous and categorical variables respectively between Groups A and B.

Preoperatively, out of 127 eyes, about half (48.8%) presented with VA > logMAR 1.0, while 28 (22%) had a presenting VA of logMAR <0.3 (Table 1). 82 were myopic (64.4%) and of those, 34 (26.8%) were highly myopic (spherical equivalent < -6.0 dioptres). 86 eyes (67.7%) were phakic and had a single tear. Upon presentation, 63 eyes (49.6%) had macula detachment, with 17 (13.4%) having proliferative vitreo-retinopathy (PVR). 90 eyes (70.9%) had 90 to <150 degrees tears, with 9 (7.1%) with extremely large tear (>180 degrees tears).

In terms of surgical procedure, two thirds of eyes underwent combined scleral buckling (SB) and trans pars plana vitrectomies (TPPV), while the other third had primary TPPV without SB **(**Table 2**)**. Of the 85 cases that included an SB, 59 (69.4%) cases had both encirclage and segmental buckling, 24 (28.2%) cases had only encirclage buckling, and only 2 (2.4%) cases had only segmental buckling. Thirty-three eyes (26.0%) had lens extraction of either phacoemulsification or lensectomy, while 28 eyes (22%) had their intraocular lens removed during the same setting. Compared to group B (1991–2005), group C (2006–2015) had significantly more cases performed with the aid of PFCL, relieving retinectomy, membrane peeling around the macula. More than half of the patients (55.1%) required either C3F8 or silicone oil.

**Table 2:** The types of retinal detachment surgeries and the intra-operative adjuncts and endo-tamponade that were utilized for the giant-retinal tear-related rhematogenous retinal detachment.

Characteristics	Group A 1991 to 2015 (n = 127)	Group B - 1991 to 2005 (n = 78)	Group C - 2006 to 2015 (n = 49)
Surgical procedure, number (%)			
TPPV	42 (33.1%)	28 (35.9%)	14 (28.6%)
TPPV + SB	85 (66.9%)	50 (64.1%)	35 (71.4%)
Lens extraction, number (% of phakic eyes)	33 (26.0%)	17 (21.8%)	16 (32.7%)
Removal of IOL, number (% of pseudophakic eyes)	28 (22.0%)	16 (20.5%)	12 (24.5%)
Intra-operative adjuncts, number (%)			
PFCL	102 (80.3%)	57 (73.1%)	45 (91.8%)
Relieving retinectomy	11 (8.7%)	3 (3.9%)	8 (16.3%)
Membrane peel	6 (4.7%)	1 (1.3%)	5 (10.2%)
Laser photocoagulation	117 (92.1%)	69 (88.5%)	48 (98.0%)
Cryotherapy	68 (53.5%)	54 (69.2%)	14 (28.6%)
Endotamponade, number (%)			
SF_6_	30 (23.6%)	22 (28.2%)	8 (16.3%)
C_2_F_6_	24 (18.9%)	8 (10.3%)	16 (32.7%)
C_3_F_8_	41 (32.3%)	29 (37.2%)	12 (24.5%)
Silicone oil	32 (25.2%)	19 (24.4%)	13 (26.5%)

**Figure 1:**
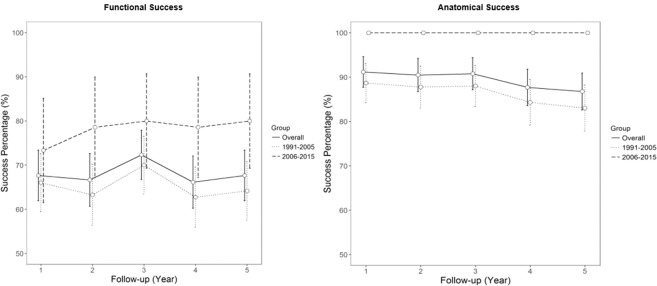
Group.  Overall. ….. 1991–2005. ----- 2006–2015.

**Figure 2:**
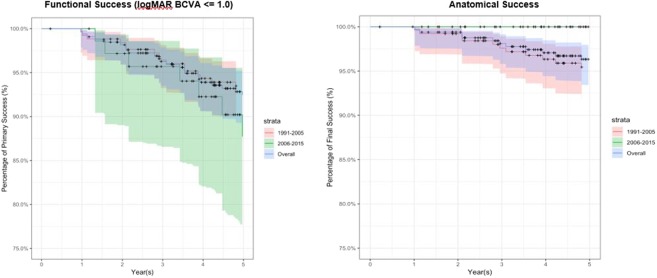
Strata.  1991–2005.  2006–2015.  Overall.

Postoperatively, the most common complication was cataract progression (n = 36, 28.3%), followed by epiretinal membrane (n = 27, 21.3%) and transient raised intraocular pressure (n = 22, 17.3%) **(**Table 3**)**. Of those, 11 (8.7%) developed proliferative vitreoretinopathy (PVR). There were no significant differences between both groups in terms of complications rates, except for a higher rate of epiretinal membrane formation in Group B compared to Group C. Only 1 out of 127 patients did not complete the post-operative 1 year follow-up. In terms of the functional outcome, the mean logMAR was 0.86 (0.89) at 1 year, and the median logMAR was 0.51 (interquartile range 0.18 to 0.51) with 88 (69.3%) achieving <logMAR 1.0 and 49 (38.6%) achieving <logMAR 0.3. For anatomical outcomes at post-operative year 1, 89 (74.8%) had primary anatomical success, while 119 (93.7%) had final anatomical success. At post-operative year 5, overall 69.3% patients still maintained <logMAR 1.0 while 87.5% with final anatomical success rate **(**Fig. 1**)**. Figure 2 shows the Kaplan-Meir survival curves for both functional and anatomical success rates between group A, B and C showing a trend towards improved anatomical and functional success rates of group C compared to B, albeit statistically insignificant.

**Table 3:** Post-operative complications, functional and structural outcomes at 1 year for giant retinal tear-rhegmatogenous retinal detachment (GRT-RRD).

Variables	Group A 1991 to 2015 (n = 127)	Group B 1991 to 2005 (n = 78)	Group C - 2006 to 2015 (n = 49)	p-value*
Functional outcome at 1 year				
Mean LogMAR BCVA at 1 year (SD)	0.86 (0.89)	0.86 (0.85)	0.86 (0.94)	0.955
logMAR BCVA < 0.30 at 1 year	49 (38.6%)	29 (37.2%)	20 (40.8%)	1.00
logMAR BCVA < 1.0 at 1 year	88 (69.3%)	52 (66.7%)	36 (73.5%)	1.00
Final Anatomical success at 1 year, number (%)	119 (93.7%)	70 (89.7%)	49 (100%)	1.00
Number of patients that required 1 or more surgeries (%) at 1 year, mean (SD)	30 (23.6%)	20 (25.6%)	10 (20.4%)	0.53
Post-operative complications, number (%)				
Transient raised IOP	22 (17.3%)	18 (23.1%)	4 (8.2%)	0.017
Glaucoma	8 (6.3%)	7 (9.2%)	1 (2.0%)	0.139
Epiretinal membrane	27 (21.3%)	10 (12.8%)	17 (34.7%)	<0.001
PVR	11 (8.7%)	7 (9.0%)	4 (8.2%)	0.381
Cataracts	36 (28.3%)	8 (10.3%)	28 (57.1%)	<0.001

*Based on chi-square or Wilcox test (categorical), or independent sample *t-*tests, comparing characteristics between Group B and C.

Close to a quarter (n = 30, 23.6%) required 1 operation or more during the 1^st^ year period **(**Table 3**)**, with 7 (5.6%) requiring 2 or more operations regardless of anatomic success. Comparing both TPPV versus SB/TPPV, the post-operative outcomes, including the complications, functional and anatomical success rate, were comparable **(**Table 4**)**. All patients with TPPV were pseudophakic. Of those, PVR is the commonest cause for re-detachment (n = 12, 9.4%). Patients with worse presenting VA, larger extent of giant retina tear (150 degrees or more), detached macula and presence of PVR were associated with BCVA of > logMAR 1.0 (Snellen VA 6/60) (all p < 0.05) **(**Table 5**)**. The types of surgery (TPPV vs combined SB/TPPV), number of breaks, lens extraction and additional cryotherapy did not have influence on the functional or anatomical success.

**Table 4:** The comparison of the post-operative outcomes between combined scleral buckle/vitrectomy versus primary vitrectomy for giant retinal tear-rhegmatogenous retinal detachment (GRT-RRD) patients.

Characteristics	All Eyes (n = 127)	SB/TPPV (n = 78)	TPPV alone (n = 49)
Post-operative complications, number (%)			
Transient raised IOP	20 (15.7%)	10 (12.8%)	10 (20.4%)
Glaucoma	8 (6.3%)	6 (7.7%)	2 (4.1%)
Epiretinal membrane	54 (42.5%)	35 (44.9%)	18 (36.7%)
PVR	12 (9.4%)	8 (10.3%)	4 (8.2%)
Visually significant cataract requiring surgery	24 (18.9%)	17 (21.8%)	7 (14.3%)
Post-operative outcomes			
LogMAR BCVA at 1 year, mean (SD)	0.85 (0.9)	0.88 (0.91)	0.82 (0.77)
LogMAR BCVA at 1 year, median (IQR)	0.86 (0.18–0.51)	0.54 (0.18–0.54)	0.54 (0.17–0.54)
Functional success at 1 year, number (%),defined as logMAR BCVA < 1.0	83 (69.8%)	53 (68.8%)	28 (70%)
Final anatomical success	109 (91.6%)	71 (92.2%)	36 (90%)
Patients who required >1 repeat surgeries required at 1 year, mean (SD)	29 (24.4%)	18 (23.4%)	11 (27.5%)

**Table 5:** Age and gender-adjusted logistic regression analysis of factors associated with functional and anatomical success at post-operative year 1.

N = 126	Functional success (logMAR BCVA < = 1.0 at 1 year)		Final anatomical success at 1 year	
	OR (95% CI)	p-value	OR (95% CI)	p-value
**Pre-operative factors**				
Race, number (%)				
Chinese	Ref	—	Ref	—
Malay	0.45 (0.08, 2.5)	0.36	NA	NA
Indian	2.73 (0.43, 53.79)	0.367	1.04 (0.12, 24.93)	0.978
Others	0.74 (0.19, 3.14)	0.661	NA	NA
LogMAR BCVA				
>1.0	0.31 (0.1, 0.84)	**0.028**	1.02 (0.2, 4.27)	0.979
0.3 < x < = 1.0	1.65 (0.41, 7.23)	0.483	3.06 (0.36, 64.65)	0.349
<= 0.3	Ref	—	Ref	—
Duration of symptoms>7 days	1 (0.99, 1.02)	0.677	1 (0.98, 1.05)	0.931
Pre-op refractive status				
Highly myopic (SE < = −6.0D)	2.44 (0.49, 11.73)	0.26	3.4 (0.37, 26.97)	0.24
Myopic (SE < 0,> −6.0D)	2 (0.42, 8.79)	0.361	5.13 (0.49, 53.04)	0.149
Non-myopic	Ref	—		
Unknown	1.6 (0.33, 7.1)	0.541	2.26 (0.24, 16.15)	0.427
Lens status				
Phakic	Ref	—		
Pseudophakic	2.38 (0.85, 7.79)	0.119	3.12 (0.52, 59.63)	0.297
Aphakic	0.94 (0.24, 4.07)	0.927	NA	NA
Extent of GRT				
90 to <120 degrees	0.85 (0.23, 2.97)	0.802	1.14 (0.14, 7.94)	0.893
120 to <150 degrees	0.57 (0.14, 2.22)	0.416	0.71 (0.08, 6.48)	0.749
150 to <180 degrees	0.24 (0.06, 0.77)	**0.022**	0.7 (0.08, 4.72)	0.713
180 degrees or more	0.09 (0.01, 0.58)	**0.017**	1 (0.07, 25.49)	0.998
Multiple tears	1.32 (0.58, 3.15)	0.515	5.96 (1.03, 113.93)	0.1
Macula status				
Attached	Ref	—		
Partially Detached	0.52 (0.11, 2.88)	0.42	NA	NA
Totally Detached	0.22 (0.09, 0.53)	**0.001**	0.37 (0.08, 1.4)	0.163
Presence of primary PVR	0.17 (0.05, 0.52)	**0.003**	2.4 (0.35, 50.92)	0.457
Operative factors				
Surgical procedure				
TPPV	1 (0.44, 2.31)	0.997	0.89 (0.25, 3.66)	0.868
TPPV + SB	Ref	—	Ref	—
Lens extraction (phakic)				
Not performed	Ref	—	Ref	—
Lensectomy	0.51 (0.2, 1.32)	0.156	0.49 (0.11, 2.27)	0.334
Phacoemulsification	0.25 (0.05, 1.2)	0.077	0.15 (0.02, 1.25)	0.051
Intra-operative adjuncts				
Heavy liquid	0.5 (0.15, 1.37)	0.203	0.9 (0.13, 3.87)	0.896
Additional cryotherapy	0.93 (0.42, 2.03)	0.85	0.42 (0.09, 1.56)	0.22

## Discussion

GRT-RRD is a relatively uncommon but complex and vision-threatening condition. This paper is the biggest (n = 127) study of over 25 years showing consistent visual acuity and anatomic stability over a 5-year post-operative follow-up period for GRT repair. Patients with worse presenting VA, larger extent of giant retina tear (150 degrees or more), detached macula and presence of PVR were less likely to achieve VA of <logMAR 1.0 by 1 year (all p < 0.05). Functional success rate was not related to the types of surgery, additional cryotherapy, number of breaks and lens extraction. The formation of cataract was found to be the most common post-operative complication, followed by epiretinal membrane and transient raised intraocular pressure. In addition, PVR occurred in approximately 8% of the patients and was the most common cause of GRT-RRD failure that required repeat operation.

The functional outcome of the patients in our study was consistent with the previous published results^2–4,8–11^ with a mean logMAR BCVA was 0.85 + 0.86 at year 1 (69.8% had logMAR BCVA better than 1.0). We note a trend of improved functional outcome over the last 10 years although the difference between both groups is statistically insignificant **(**Fig. 2**)**.

The GRT-RRD patients also had good anatomical success with 91.3% retina attachment rate and a trend towards improved results seen over the past 10 years. Our study has comparable success rates to previous published studies with reported anatomical success rates ranging from 65.5% to 95%^3,4^. No other study has reported these outcomes of up to 5 years of post-operative follow up. We postulate that this trend towards better outcomes in the latter group could be due to mainly to the availability and use of PFCLs, and also improved vitreoretinal surgical instrumentation (higher cut speed, the cutter being nearer the tip that allows better shaving), the advent of minimally invasive vitrectomy surgery for better intraocular stability and wide-angle viewing systems. In addition, there was a trend towards lens or IOL removal, enabling better visualization and access to the vitreous base, especially at the area of radial extensions. With planned lens extraction, this facilitates preservation of the lens capsule for future secondary intraocular lens implantation, especially for eyes that require silicone oil endotamponade. Importantly, the stability of the anatomical and functional outcomes persisted over a 5-year period for our patients, similar to reports from earlier smaller studies^12,13^.

Several earlier studies have also reported successful repair of GRT-RRD using TPPV alone without the addition of SB^2,14^. In our cohort however, two thirds of our patients underwent combined SB/TPPV and all were phakic. The placement of additional SB was to provide additional support to the vitreous base and for ora occlusion. Both surgical methods are shown to be comparable for post-operative complications, functional or anatomical success in our study. The role of SB placement is to support the vitreous base, reduce the circumference of the globe, relieve the vitreo-retinal traction and provide a more thorough ‘shaving’ of the vitreous base^15^ consistent with what Goezinne *et al*.^16^ and Verstraeten *et al*.^17^ suggested. The placement of SB could potentially cause posterior retinal slippage by changing ocular contour and scleral shortening relative to retina, gaping of retinal tissue, redundant retinal folds when the buckle is tightened and fish-mouthing^11,14^; all of which were not reported in our study.

There was an increased use of PFCLs in our study throughout the 25 years possibly due to several reasons. First, it is favoured as an intra-operative tamponade in view of their biologically inert property and ensures good apposition of the entire retinal tear to the underlying retinal pigment epithelium (RPE). It can also displace subretinal fluid and blood, allowing a more effective laser retinopexy to create better chorioretinal adhesion^4,7^. It may also reduce the incidence of inferior PVR by preventing the pooling of RPE cells, chemoattractants and serum components over the inferior retina^3^. Concurrently, there was a decrease in the application of cryopexy, given the advent of the endoilluminated lasers enabling better visualisation of the anterior edge of the GRTs. Epiretinal membrane was reported at a significantly higher rate after GRT repair in the latter compared to the former group. This could possibly be due to an increased usage of macula optical coherence tomography and its sensitivity in evaluating the retina compared to earlier years.

The strengths of our study include a large sample size collected over 25 years, with comprehensive pre-, intra-, postoperative details and reporting one of the longest follow-up periods of outcomes. The limitations include the retrospective nature and only about a 60% of patients who completed the 5-year follow up. In addition, the diagnosis of GRT was made based on the surgeon’s diagnosis from the electronic medical records, with no confirmation on the wide-field retinal fundus photography. We did not possess any information on the location of the retinal tear in our study, and future research is of great value to analyse this as another risk factor for retinal re-detachment.

In conclusion, our study shows an improved trend of GRT-RRD functional and structural outcomes over the past 25 years with good and stable functional and anatomical outcomes. Worse presenting VA, larger extent of giant retina tear (150 degrees or more), macula-detached status and presence of PVR were associated with poorer visual outcomes. These factors would help with the pre-operative counselling and intra-operative surgical decisions.
